# Flexural Properties in Edgewise Bending of LVL Reinforced with Woven Carbon Fibers

**DOI:** 10.3390/ma16093346

**Published:** 2023-04-24

**Authors:** Neda M. Sokolović, Ivana Gavrilović-Grmuša, Vladislav Zdravković, Jelena Ivanović-Šekularac, Darko Pavićević, Nenad Šekularac

**Affiliations:** 1 Faculty of Architecture, University of Belgrade, 11000 Belgrade, Serbia; 2 Faculty of Forestry, University of Belgrade, 11000 Belgrade, Serbia

**Keywords:** laminated veneer lumber, CFRP, reinforcement, epoxy adhesive, melamine-urea formaldehyde, mechanical characteristics, bending strength, bending modulus of elasticity

## Abstract

This paper presents the results of experimental testing of the bending strength and modulus of elasticity in edgewise bending of unreinforced and reinforced seven-layer LVL (laminated veneer lumber) poplar veneer panels. The aim of the research is to determine the influence of woven carbon fibers on the improvement of the bending properties and modulus of elasticity of LVL bending in the plane of the plate, as well as the influence of adhesives on the bending properties of the composite product, in order to test the potential of using this newly obtained material as a structural element. Bending was performed on small-scale samples. The main research task is the examination of three types of reinforcement, which differ from each other in position, orientation, and number of layers of reinforcement, using two different types of adhesives: epoxy adhesive and Melamine Urea Formaldehyde Resins (MUF). The composite material was produced in four different combinations in relation to the orientation and position of the reinforcement in the layup. The applied reinforcement is defined through three different configurations (EK1, EK2, and EK3) and a fourth control sample (EK4). Each configuration was produced by applying the two previously mentioned types of adhesives. The research findings showed that in the case of samples produced by applying CFRP (carbon fiber reinforced polymer) using epoxy adhesive, it significantly affected the increase in bending strength and flexural modulus of elasticity. The average improvement in bending strength is 32.9%, 33.2%, and 38.7%, i.e., the flexural modulus of elasticity is 54.1%, 50.7%, and 54.7%, respectively, for configurations EK1, EK2, and EK3, compared to control sample EK4. During the testing, the test samples from reinforced panels EK1 and EK2 showed partly plastic behavior up to the fracture point, while the diagram for the test samples from reinforced panels EK3 shows elastic behavior to a considerable extent, with a significantly smaller plastic behavior zone. This research proved the impossibility of using melamine-urea formaldehyde adhesive to form a composite product based on veneer and carbon fabric. The greatest contribution of this work is the experimentally verified and confirmed result of the possibility of applying poplar veneer to design structural elements in LVL using epoxy adhesive.

## 1. Introduction

In practice, timber constructions are often combined with other materials to improve their mechanical properties and increase the structural member capacity. Primarily, this is a consequence of the conservation of timber structures, where a coupled load-bearing structure is formed using another material. The earlier practice of restoration and strengthening of timber members was mainly based on the application of metal-reinforced timber, while in the last few decades, fiber-reinforced polymers have become much more prevalent. The idea of strengthening with synthetic materials is not new and has been the subject of many research studies since the 1960s [1]. The pioneers of strengthening timber structures using polymer composites were Wangaard and Biblis [2], who were the first to carry out the strengthening of timber with polymer materials using glass fiber reinforced polymers (GFRP).

Veneer-based composite products were not the object of rehabilitation, i.e., subsequent strengthening of structural elements during the building’s exploitation. Nevertheless, the topic of improving the structural characteristics of these products during production, especially plywood using fiber-reinforced polymer, appeared for the first time in the 1970s, when the APA (American Plywood Association) for the first time applied GFRP to test the strengthening of plywood [2]. The testing included uni-directional fibers, bi-directional woven roving, and chopped-strand mats with vinyl ester and polyester resin. Testing of flexural strength, stiffness, and impact resistance showed that plywood reinforced in some of the mentioned ways could be used in the transport industry. More intensive research in this area was carried out again in the early 2000s when Biblis and Carino examined the bending properties of three- and five-layer plywood reinforced with glass fiber fabric using phenol-based adhesives [3].

This type of reinforcement provides the possibility of creating a new structural material, a composite material with pre-planned mechanical characteristics. Its properties were pre-designed in relation to the needs that the material should satisfy in construction. In addition, the advantage of this method of strengthening is the possibility of structural application of low-mechanical-property wood veneers. As well as the application of technically weaker wood, especially local species such as poplar, which is not generally used as structural timber. Serbia is the fifth country in Europe in the area of poplar plantations [4], which is also one of its primary resources in the wood industry. However, its lower load-bearing capacity and low mechanical properties limit its application in structural products.

In order to increase the use of poplar wood in construction, its mechanical properties are improved through lamination, that is, the formation of veneer-based products in the form of plywood and LVL panels. The improvement of the mechanical properties of these products is also tested by combining poplar veneer with veneers of other types of wood [5,6,7] or with some other synthetic materials. Engineering wood products based on poplar veneers and composite materials have great potential for structural use [8]. Combining veneers of technically soft wood with fiber-reinforced polymers can produce a product of uniform quality with better physical and mechanical characteristics. This ensures better utilization of raw wood materials. That can be a particular benefit to the wood industry and the production process of wood-based products, as it impacts the economic profitability and appropriate utilization of wood reserves, which is both economically and environmentally beneficial.

Laminated veneer lumber (LVL) is a wood product similar to plywood, composed of layers of veneer, but unlike plywood, LVL is composed of mostly parallel grain laminations of veneer [9,10,11], i.e., there can be up to 20% layers perpendicularly oriented. Due to its good mechanical properties, dimensional stability, and easy workability, LVL is used both as a structural and non-structural material. Its most common application in the form of structural material is as chords of trusses loaded for tension or as a valuable material for flanges of I-beams [11] or beam elements. The great potential for using these products for structural use is in the unconventional method of application—spatial structures designed from LVL or plywood panels. Wood-based panels form the entire structure, becoming the primary supporting element in spatial structures, primarily polygonal shells, and folded structures [12]. Panels in these structures were exposed to stress from axial forces in the plane of the panels. During the action of the force in the plane of the panel, some elements of these structures are exposed to edgewise bending. Often, plywood and LVL panels in this type of structural use have some disadvantages, and some mechanical properties must be increased.

However, despite its great potential, LVL produced from poplar wood was not often applied as a structural element, precisely because of the low mechanical properties of the wood raw material itself. According to all of the above, this research will focus on the flexural properties of poplar LVL panels reinforced with CFRP exposed to force in the plane of the panel. Hypotheses can be made based on the previously observed observations and read as follows: by strengthening panels based on poplar veneer by inserting carbon woven fabric between the veneer sheets, the mechanical characteristics of LVL panels are improved, and as such, they can be used as primary structural elements.

### 1.1. The Research Subject and Objectives

The advantages of poplar wood are reflected in its small density, simple processing, easy reproducibility, and low raw material prices. The main disadvantages of using it for the design of wood structural elements are precisely its low mechanical properties as well as its large variation. The fast-growing poplar wood is most often used for the production of non-structural plywood, primarily as a decorative element of interior cladding, as well as in the packaging industry. In recent decades, this wood has been increasingly represented as a raw material for the production of wood-based materials, especially wood-based products (veneer and OSB (Oriented Strand Board) panels, LVL and CLT (Cross-Laminated Timber) beams, etc. The paper focuses on improving the structural properties of panels based on poplar veneer and, above all, on experimental testing of the structural properties of composite materials based on poplar and CFRP.

This research investigates the possibility of producing composite panels based on veneer reinforced with CFRP. The focus is on the investigation of the possibility of applying this material as structural elements in architectural buildings, especially those exposed to forces in the plane of the plate, like beams, polygonal shells, and folded structures.

Special attention is focused on determining an effective method of strengthening and an adequate choice of adhesive. The research includes two different adhesives in terms of chemical composition and method of synthesis and cross-linking: epoxy (polyaddition cross-links without extracting nus products) and MUF (polycondensation with extracting small molecules of water and formaldehyde).

After producing the samples, the tests are focused on the analysis of the behavior of the bending properties of the reinforced panels. The aim of the paper is to point out the potential of using poplar as a structural material.

### 1.2. Literature Review

An examination of the influence of the amount of carbon fibers in the strengthening of plywood with CFRP products on its mechanical properties and, above all, on its bending properties was presented in research by Brezović et al. [13]. The bending properties are perpendicular to the plane and the shear properties of the reinforced five-layer plywood were tested. Reinforced plywood showed an increase in bending strength of 30–40% and a flexural modulus of elasticity of 80–140% compared to the control panel. A greater amount of synthetic fibers in the matrix affects the reduction of stress and deflections in the composite product, which positively affects the panel’s bending properties. Apart from the quantity, research also focuses on the influence of the orientation angle of carbon fibers on the bending and shear properties of the five-layer poplar plywood [14], as well as the length and orientation of the synthetic fibers on its bending properties [15].

Strengthening of veneer-based panels with carbon fibers oriented in two directions and analysis of their position in construction on the mechanical properties of the plywood [16] shows an increase in flatwise bending strength, tensile strength, and Young’s modulus of elasticity. However, it also shows that the best results in increasing the tensile strength in the transverse direction are achieved when the synthetic fibers are placed closer to the neutral axis of the panel. Examination of the mechanical properties of five-layer poplar plywood reinforced with GFRP and glued with PF (Phenol Formaldehyde) adhesive [17] showed an increase in the specific modules of elasticity and specific module of rupture, but also a decrease in the shear strength of the reinforced samples compared to the control sample. By testing the flexural properties of reinforced LVL panels [18], it was proven that glass fibers oriented in two directions contribute to an increase in modulus of rupture and modulus of elasticity when bending perpendicular to the plane of the sheet, but that the increase compared to the control samples is much more pronounced when bending in the plane of the panel. This is the result of an equal distribution of stress through several layers of veneer and fiberglass, not just through the last layer in the tensioned section zone. For the same samples, flexural properties under dynamic loads were tested [19], which showed that LVL reinforced with glass fibers contributes more to impact bending strength under dynamic compared to static loads. Examining the physical properties of these panels [20] showed that some physical properties are better in reinforced panels than in non-reinforced ones, which may result from a larger amount of adhesive applied. Overall, better physical properties, as well as dimensional stability, are the result of both the applied fibers and the greater amount of adhesive, as well as its penetration into the wood structure.

Despite intensive work to change the current situation in the wood industry by introducing eco-friendly adhesives, the fact is that formaldehyde-based adhesives represent 90% of all adhesives in use. Some research is aimed at the use of formaldehyde adhesives for creating composite structures. They show that it is possible to improve flexural properties, especially modules of rupture and elasticity, by reinforcing five-layer plywood with CFRP using MUF adhesive [21].

Testing the bending properties of eleven-layer plywood produced from poplar, eucalyptus veneer, or their mutual combination reinforced with carbon fabrics with fibers oriented in one direction [22] shows that the increase in the module of elasticity and the modulus of rupture in the longitudinal direction is significantly influenced by the positioning of the reinforcement on the outer surfaces of the composite product. Positioning reinforcement in the core increases the modulus of elasticity and the modulus of rupture in the transverse direction of the plate.

The investigation of the effect of the amount of fibers on the buckling of GFRP-reinforced plywood under exposure to compressive stresses and their behavior under exposure to different temperature conditions was conducted by Choi, S.W. and a group of authors [23,24].

Analyzing the process of producing boards based on poplar veneer using a multi-stage hot pressing system and analyzing the mechanical properties of these boards [25], reinforced with glass fibers, carbon fibers, or their combination, show that the best behavior under the influence of load and the greatest improvement of mechanical properties have the products formed by the combination LVL + GFRP + CFRP. Furthermore, multi-stage pressing allows the adhesive to better penetrate the veneer layers and form stronger bonds between the layers.

In addition to testing LVL, the mechanical properties of laminated veneer lumber beams strengthened with FRP materials [26,27,28], especially the flexural capacity of LVL reinforced with CFRP [29,30,31,32], as well as shear and compressive stress [33], were also tested.

## 2. Materials and Methods

### 2.1. Veneer Preparation

In this study, constructive poplar veneers, produced by peeling (rotary-peeled veneers) at a thickness of 4 mm and humidity of 7 ± 1%, manufactured by “VisaProm” doo, Kanjiža, were selected for the formation of LVL panels. All veneers are cut to dimensions of approx. 550 × 550 × 4 mm. The classification and selection of veneers suitable for panels are determined by visual observation of the veneers. First-class veneers were selected, without knots or other defects in the wood. Reinforced and control LVL panels were formed from all veneers. Two sets of sheets were formed, the first using epoxy adhesive (EK panels) and the second using melamine-urea formaldehyde adhesive (MK panels). In each set, three types of reinforced sheets and one type of non-reinforced sheet were formed. LVL panels were produced in the laboratory of the Faculty of Forestry.

### 2.2. Adhesives

To form the first set of panels, epoxy adhesive “MapeWrap 31” was applied in all layers, between the veneers as well as between the veneers and the FRP. The adhesive was formed in a mass ratio of A:B = 4:1; it was applied on one side of the veneer in an amount of 250 g/m^2^ to ensure that the specified amount of adhesive was in the glue line between the two layers of veneer. The same amount of adhesive was applied to each layer of CFRP wood on both sides of the carbon fabric.

The second set of panels was formed using melamine-urea formaldehyde adhesive (MUF) “AkzoNobel” 1247/2526. The adhesive was made in the mass ratio A:B = 5:1 and was applied in the same amount and in the same way as the previously described epoxy adhesive. The technical properties of the adhesive are shown in Table 1 [34,35]. The technical properties of MUF adhesive were experimentally tested according to the standard [36]. The gel time for epoxy adhesive was experimentally examined.

### 2.3. Woven Carbon Fiber

The unidirectional “plain-weave” type of knitting carbon fiber, MapeWrap C UNI-AX 300/40”, weighing around 300 g/m^2^, was used for reinforcement. The physical and mechanical properties of the CFRP fabric are shown in Table 2 [37].

### 2.4. Manufacturing LVL

**Figure 1:**
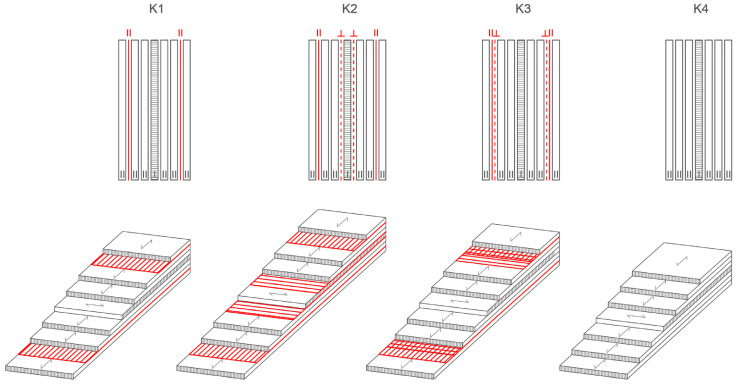
Strengthening configuration of reinforced and unreinforced LVL panels.

All panels are produced as seven-layer panels, with six veneer sheets oriented in the longitudinal direction and the central, fourth veneer sheet oriented perpendicular to the grain of the outer veneer sheets. Reinforcements are placed between the veneer layers according to the following scheme, shown in Figure 1:

- Combination 1 (K1)—reinforcement placed in the first and sixth gluelines (S1║6║), unidirectionally oriented, carbon fibers placed longitudinally, parallel to the grain of the outer veneer;
- Combination 2 (K2)—reinforcement placed in the first and sixth gluelines, parallel-oriented as outer veneer, and the third and fourth gluelines, perpendicular to the direction of orientation of the outer veneer sheets (S1║3┴4┴6║);
- Combination 3 (K3)—reinforcement placed in two layers perpendicular to each other, forming a mesh in two directions. The fabrics are placed in the first and sixth gluelines (S1║┴6┴║), taking into account the symmetry of the cross-section;
- Combination 4 (K4)—unreinforced panels, i.e., control reference samples.

**Figure 2:**
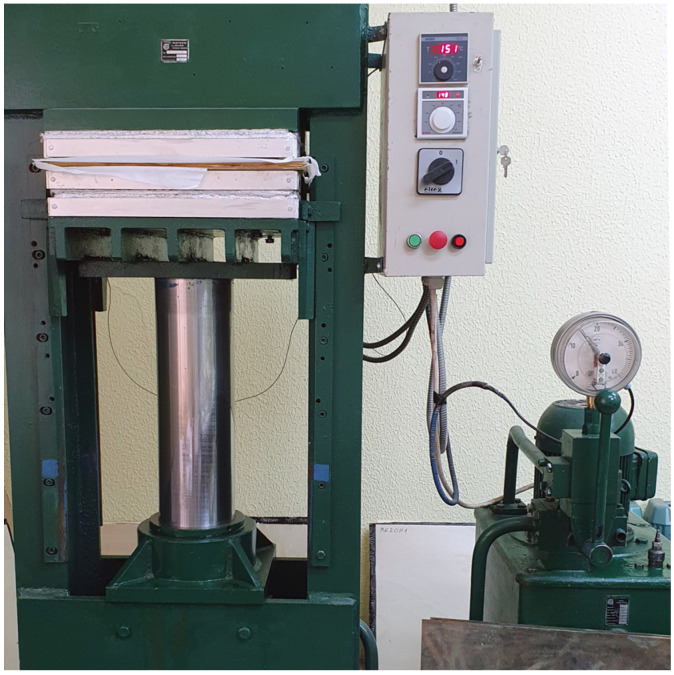
Process of pressing LVL panels in a laboratory press.

**Figure 3:**
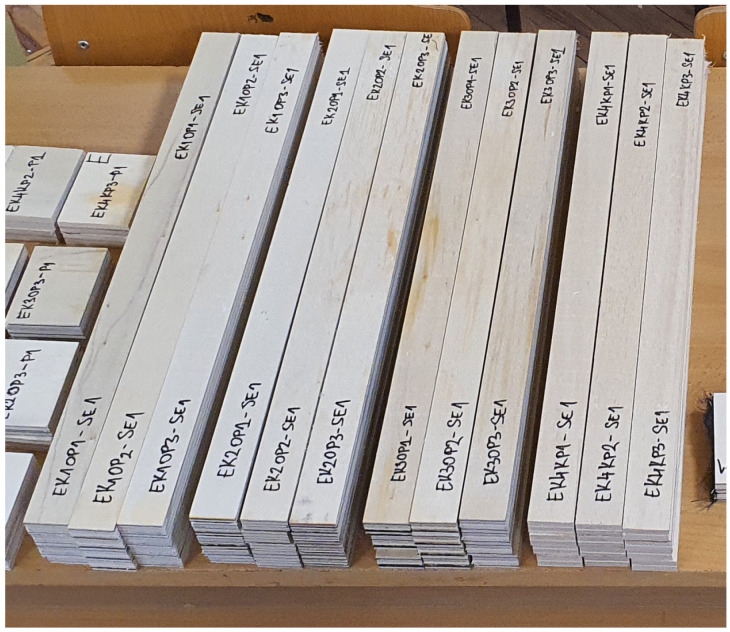
Samples for edgewise bending test.

For each combination, six panels measuring approx. 55 × 55 cm were formed (3 LVL panels using epoxy adhesive and 3 LVL panels using MUF). All panels were pressed in a laboratory press for 15 min from the moment of reaching a temperature of 100 °C in the central layer at a pressure of 1.5 MPa (Figure 2). After that, they were stored for a week to cure. After conditioning, 30-mm edges were trimmed off of the panels, and samples were cut from the panels. Figure 3 shows epoxy samples for the edgewise bending test after cutting the panel. Samples were formed in accordance with standards SRPS EN 14374:2012 [38], SRPS EN 408:2014 [39], SRPS EN 789:2012 [40], SRPS EN 325:2014 [41], and SRPS EN 322:2010 [42].

**Figure 4:**
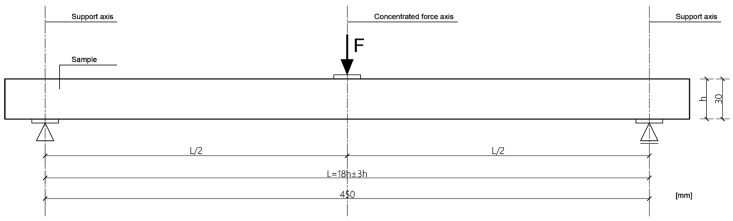
Schematic of the test setup.

Bending strength and modulus of elasticity in bending (MOE) tests were done in the edgewise direction, as shown in Figure 4 (three-point bending). All test samples were conditioned in a climatic room at 20 ± 1 °C and 65 ± 5% relative humidity prior to the tests. Twelve test samples with dimensions of $520\times 30\times t\;\left(\mathrm{mm}\right)$ for bending strength, and MOE were prepared for each group and each adhesive. The average thickness of panels with epoxy adhesive was t≈14.5 mm, and for MUF t≈11 mm. Test samples were cut from boards parallel to the grain.

### 2.5. Tests of Mechanical Properties

**Figure 5:**
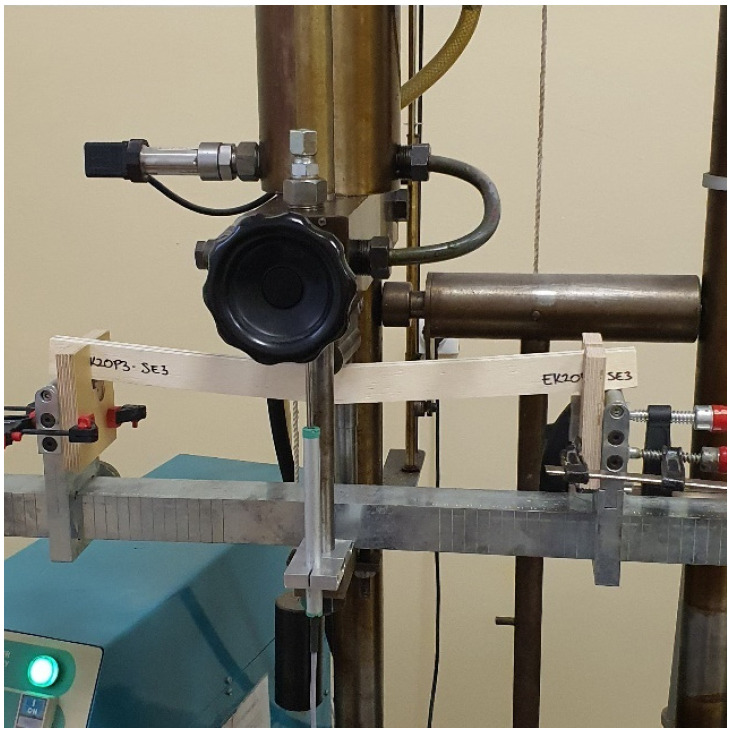
Testing of bending strength and the module of elasticity in edgewise bending test.

The mechanical properties of the panels were tested at the University of Belgrade, Faculty of Forestry, at the chair of primary wood processing, in the laboratory for wood properties testing. The test was performed on a machine for testing the mechanical properties of wood and wood products, “Wood Tester WT-4”, with a maximum force capacity of 40 kN. The bending tests were carried out in accordance with the requirements of the standards SRPS EN 14374:2012 [38] and SRPS EN 408:2014 [39]. According to the standard mentioned, the samples are tested as beams loaded with two concentrated forces in the middle of the span. The test deviated from the standard in the way the load was applied, and instead of the four-point static bending test, a three-point static bending test was performed within the scope of this work, as shown in Figure 3. A total of 96 prismatic samples with cross-sectional dimensions of 30 mm × t were tested, and the span between support points was 450 mm. The standard defines the range between support points and is l = 18 h ± 3 h (Figure 4). The deflection was measured in the middle of the beam using an inductive deflection meter. The modulus of elasticity was measured using a probe for testing the modulus of elasticity. The test lasted until failure with a controlled displacement speed of 2.0 mm/min, and thus the maximum force was reached in an interval of 300 ± 120 s, as defined by the standard. Registration of data on the maximum force, modulus of elasticity, and maximum deflection was recorded through the acquisition system “Console”. After the tests, the failure mode was recorded. Figure 5 shows the procedure for testing one sample.

## 3. Results and Discussion

### 3.1. Bending Behavior and Failure Mode

#### 3.1.1. Epoxy Adhesive Samples (EK)

**Figure 6:**
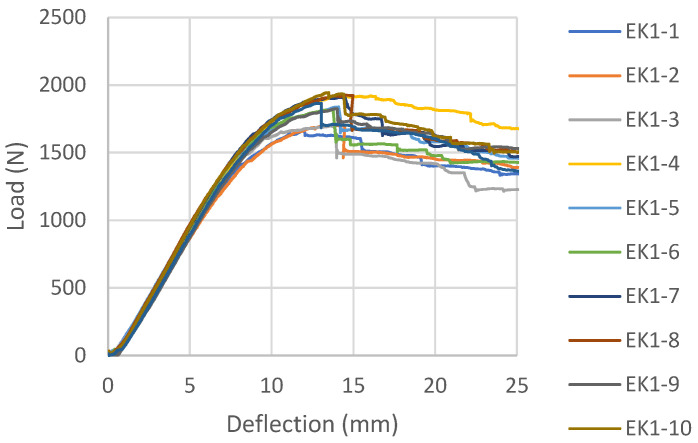
Load-deflection diagrams designed for EK1 beams.

**Figure 7:**
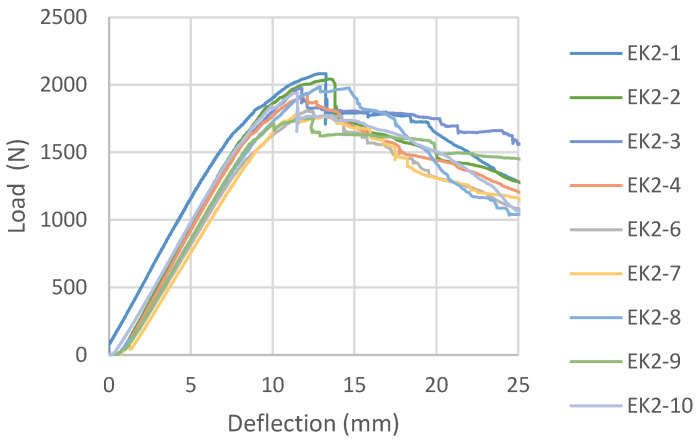
Load-deflection diagrams designed for EK2 beams.

**Figure 8:**
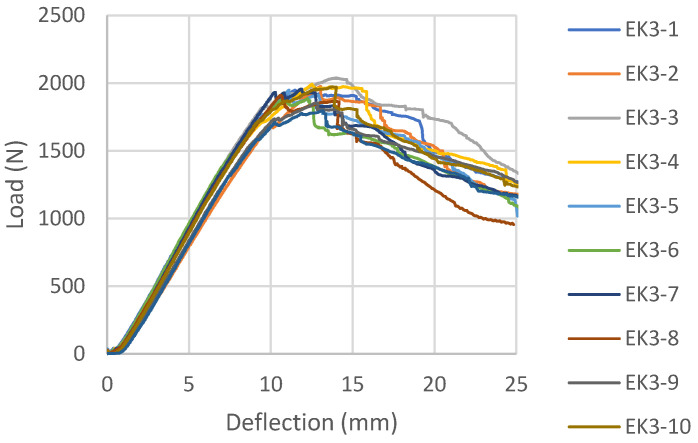
Load-deflection diagrams designed for EK3 beams.

**Figure 9:**
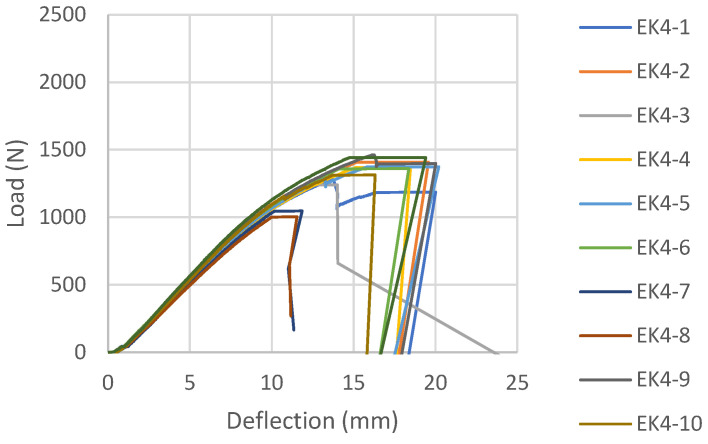
Load-deflection diagram designed for EK4 beams.

Load-deflection diagrams for all tested samples glued with epoxy adhesive, according to the reinforcement combinations, are shown in Figure 6, Figure 7, Figure 8 and Figure 9. During the testing, all ten samples per combination of loads, glued with epoxy adhesive, showed good behavior at edgewise bending tests. All reinforced samples showed similar behavior during bending.

**Figure 10:**
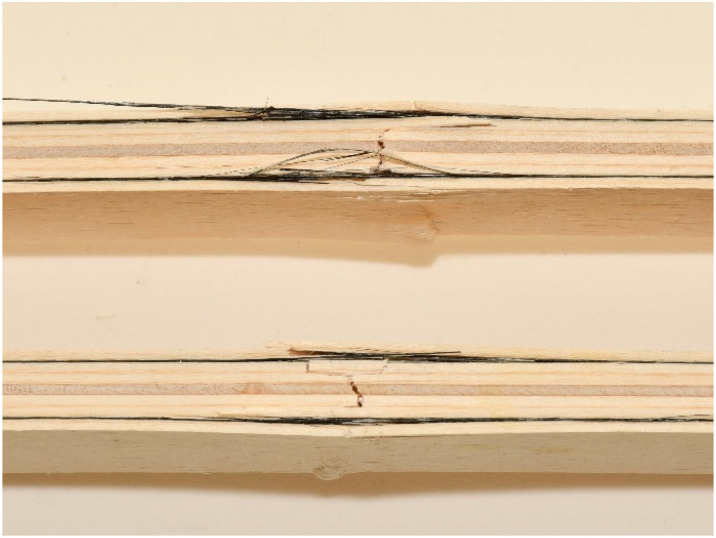
EK1 samples failure from edgewise bending: wood layer cracking and fiber tearing.

**Figure 11:**
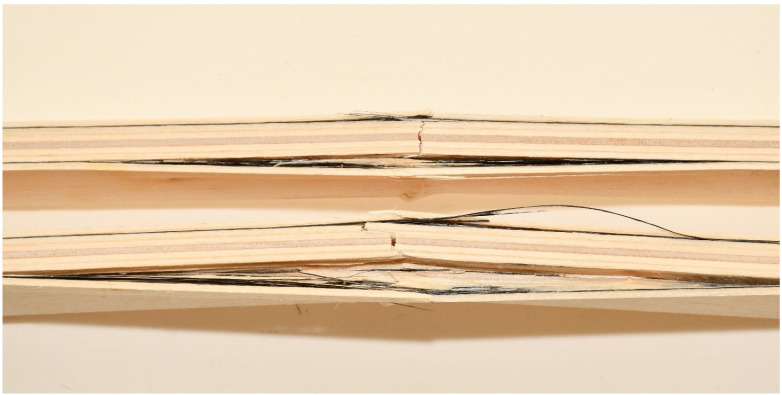
Substrate failure sampling for EK1OP2-SE3 and EK1OP3-SE3.

The samples strengthened according to the combination EK1 show a certain ductile behavior. The zone of the non-linear behavior of the test samples is somewhat shorter than expected. The plastic flow of the composite was interrupted by the beam failure, which occurred in the tensioned zone of the cross-section in all test samples. In all tested combinations, the damage occurred in the middle third of the beam span, at the load-bearing point. The failure of all test samples occurred through the veneers, i.e., in the central layers, between two carbon fabrics. A typical beam fracture for the EK1 combination is shown in Figure 10. The fracture of all samples occurs in the central layer in the tensioned zone of the test sample and moves towards the outer layers, followed by the tearing of a very small number of carbon fibers. The adhesion between the wood and the reinforcement was effective until the failure. After the fracture, in some samples, the delamination of the carbon fabric from the wood occurs, as well as the splitting of the veneer layer along the substrate in the longitudinal direction. One part of the wood remains attached to the carbon fabric, while the other part remains in the middle zone of the panel, which is shown in Figure 11.

In EK2 samples, the layers of carbon fabric located in the third and fourth gluelines are placed in the direction of the force and have no influence on the reinforcement of the panel, as expected for this mechanical property. They do not contribute to changing the composite product’s behavior under load, as shown by the load-deflection diagram and its similarity to the EK1 diagram. The material’s behavior is almost identical to that of the previously shown combination, except for some ductile behavior. For the most part, the diagram shows a linear trend, followed by plastic behavior interrupted by failure. The damage to all EK2 test samples also occurred in the central third of the span, with a brittle break of the central layers of the beams, as in the case of EK1 samples.

The load-deflection diagram of the sample EK3 (Figure 8) shows a distinctly linear elastic behavior until failure, which is not the expected behavior for an LVL reinforced with carbon fibers. In this combination, the fibers are placed in the first and sixth gluelines in two layers perpendicular to each other, creating a bi-directional carbon mesh. Apart from the fact that the zone of plastic behavior before fracture is almost negligible, it is noticeable that the force intensity decreases in certain test samples after fracture, increases again, and then permanently decreases.

**Figure 12:**
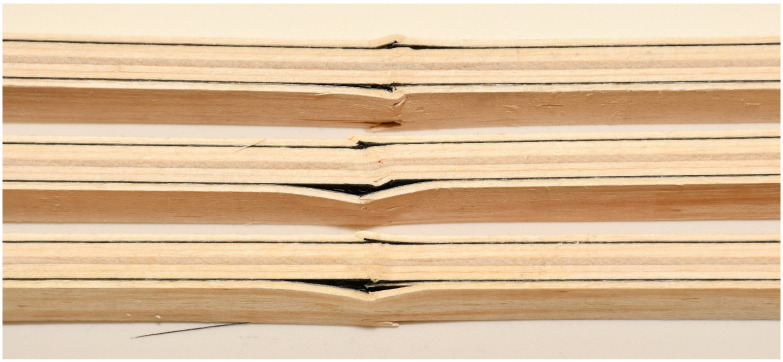
Failure of test samples EK3 in the pressure zone, crushing of fibers, and delamination in the FRP-FRP layer.

In this combination, the system is wood-glue-CFRPǁ-glue-CFRPꞱ-glue-wood which does not have the same behavior as the wood-glue-CFRPǁ-glue-wood system. Stress in the lined part of the diagram of EK3 samples was taken over by the CFRPǁ-glue-CFRPꞱ layer, which is also twice as thick as the individual layers of carbon in other combinations. The brittle epoxy adhesive between the two layers of carbon fibers contributed to almost linear deformations. After the break in this layer, the force dropped sharply, so the inner plies took over the stress until the final failure. The final failure of all the test samples was on the wood, in the central part of the support, in the line of the load position. Carbon fiber breaks in these samples seldom occur. The carbon fibers formed into a bi-directional mesh assumed the role of a supporting layer, and even after the test sample broke on the wood in the tension zone, there was an increase in force, but this growth was interrupted by the cracking of a smaller layer of fibers and the outer veneer sheets in the tension zone. In the section’s upper zone, the compressive strength was exceeded, followed by the wood fiber buckling in the plane. That occurred after the cracking of the wood in the tensioned zone. The result of this damage is delamination in the reinforced FRP-FRP layer, as well as in-plane and out-of-plane wood fiber buckling. This failure is shown in Figure 12. In all the tested samples made of reinforced panels, the fracture in the wood was brittle and explosive in nature. Adhesion failure in the stressed zone between the FRP-wood and FRP-FRP layers in the reinforced panel test samples occurred exclusively after the failure in the wood.

**Figure 13:**
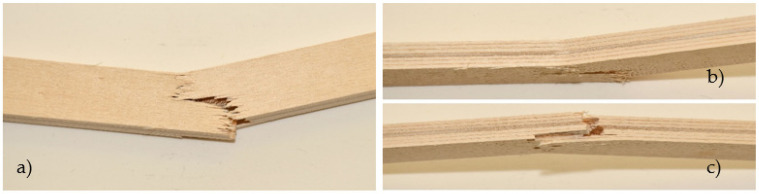
Control unreinforced test samples EK4: (**a**) fracture of the test sample; (**b**) crushing by exceeding the applied pressure (**c**) tension zone fracture.

Control samples of EK4 panels show more ductile behavior than reinforced panels (Figure 9), which is not expected. The plastic behavior was interrupted by the collapse of the samples, which was caused by a brittle fracture in the tensioned section zone. The fracture is the same in all tested samples of Combination 4. The fracture is sudden and explosive, and unlike the strengthened samples, which remain compact, it cuts through all the wood fibers, as can be seen in Figure 13.

**Figure 14:**
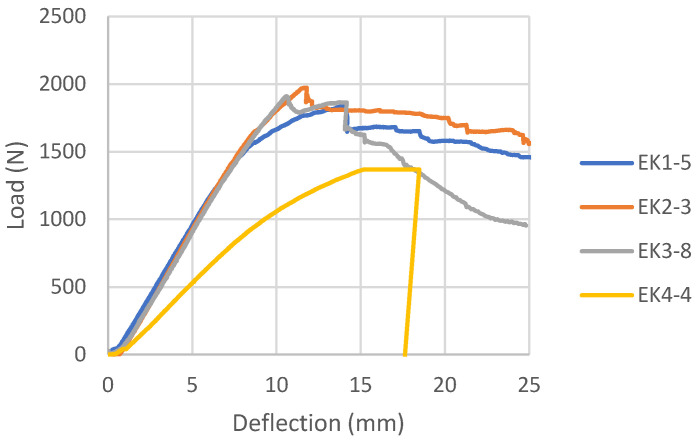
Comparative load-deflection diagram for epoxy samples.

The behavior of characteristic test samples for each combination of panels is shown in Figure 14 through a comparative diagram. This diagram provides a clearer overview of all the above. All reinforced samples have plastic behavior, and the control sample (EK4) has typical behavior for LVL panels. This sample has expressed ductile behavior, while the above can be noted in a small part in sample EK1.

#### 3.1.2. Melamine-Urea Formaldehyde Adhesive Samples (MK)

**Figure 15:**
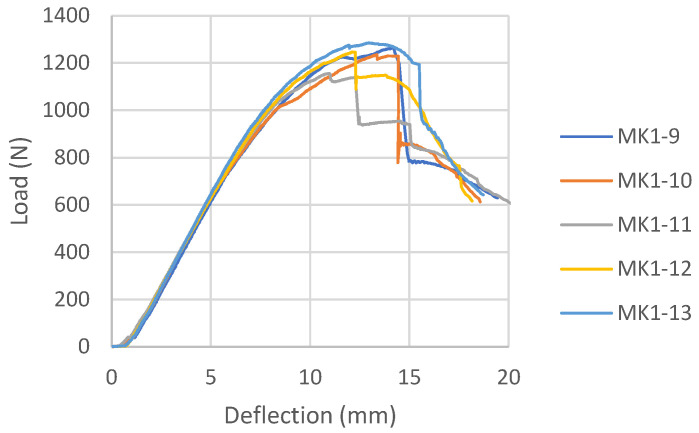
Load-deflection diagram for MK1 samples.

**Figure 16:**
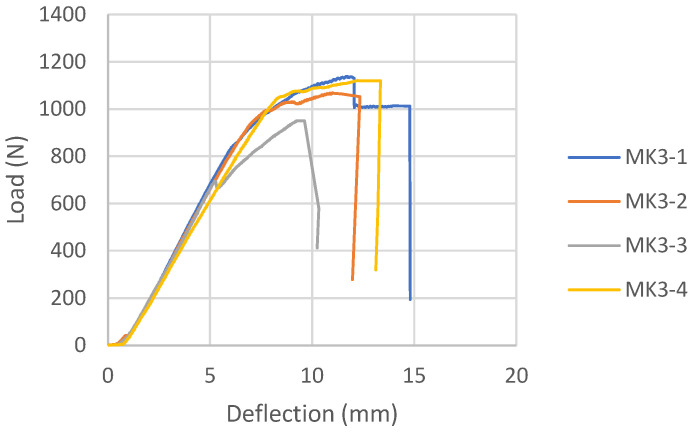
Load-deflection for MK3 samples.

**Figure 17:**
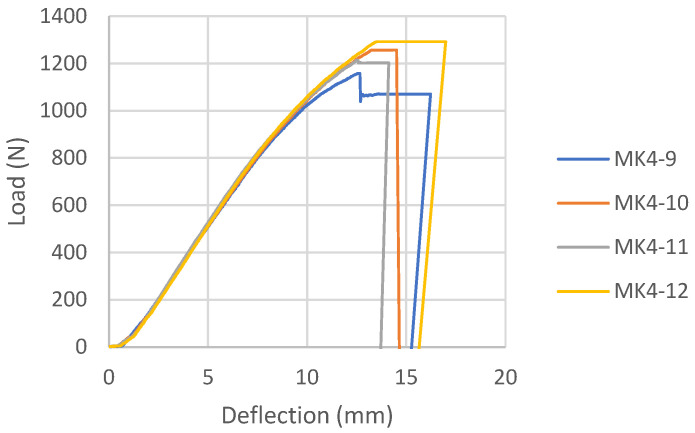
Load-deflection diagram for MK4 samples.

**Figure 18:**
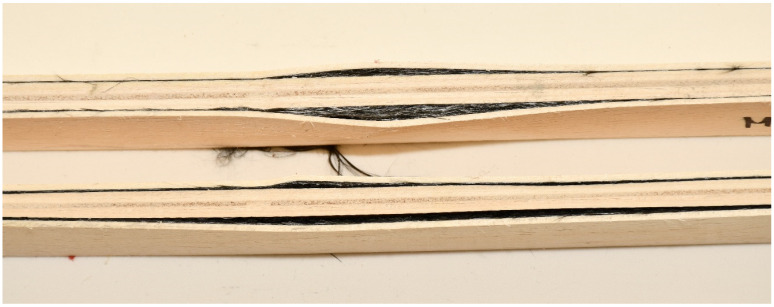
Delamination in the FRP-FRP layer in the test samples from MK3 panels after testing.

The load-deflection diagrams for all tested samples bonded with MUF according to the reinforcement combinations are shown in Figure 15, Figure 16 and Figure 17. During the examination of panels bonded with MUF, it was observed that during loading, most often at an intensity of 70% of the maximum force, before the excessive force initiates the test sample fracture, delamination of the test samples occurs between the FRP-FRP layers in MK3 panels (Figure 18), the FRP-wood layer and the FRP-FRP layer, starting in the middle of the stressed zone of the support, at the point of force action, and continuing along the entire stressed zone. After that, the test sample continues to bear the load; it breaks properly in the tensioned section zone in the middle third of the span, but its entire load capacity is reduced to the middle layers of the veneer. In Combination MK1, it behaves like a five-layer panel, ignoring two layers of veneer and carbon fibers. In Combination MK2, delamination occurs in the FRP-wood layer, both in the core and outer layers.

There will be no mention of the types of failure of the combinations shown because the failure cannot be defined as a composite product failure. Before the fracture itself, the delamination of the reinforcement and the outer layers of the veneer from the central part of the section occurred, and the element ceased to behave as a composite material.

The load-deflection diagram for MK1 samples (Figure 15) shows the ductile behavior until failure, which is significantly more ductile than the same combination of reinforcements for panels bonded with epoxy adhesive. The load-deflection diagram for MK3 samples (Figure 16) shows the collapse due to brittle fracture, which is a characteristic of an unreinforced test sample. The behavior of the MK4 control panel bonded with MUF (Figure 17) on the load-deflection diagram shows similar behavior to the control panel bonded with epoxy adhesive.

### 3.2. Load-Bearing Capacity

#### 3.2.1. Epoxy Adhesive (EK) Samples

**Figure 19:**
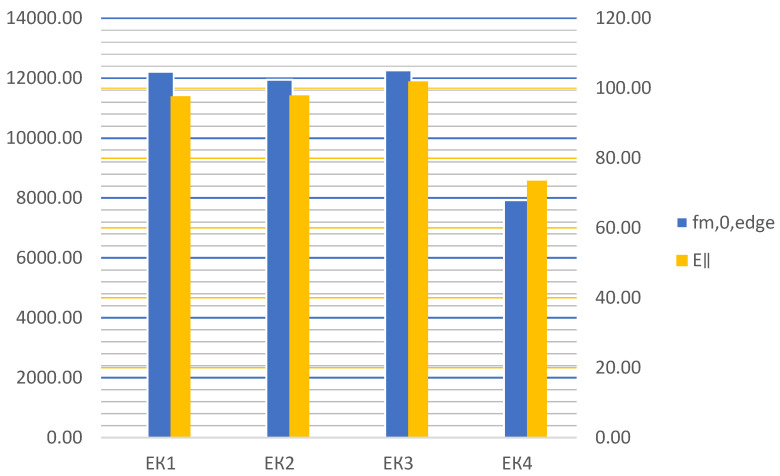
Diagram of values $f_{m,0,edge}$ and $E_{∥}$ under different reinforcement.

The test results for the test samples are shown in Table 3. Taking into consideration samples bonded with epoxy adhesive, compared to non-reinforced samples, cracking occurs at significantly higher loads in reinforced ones. In terms of percentage, the increase in the bearing capacity of the reinforced beams compared to the control samples ranges from 40.7% for beams EK1 to 48% and 48.1% for beams EK2 and EK3. The bending strength capacity is the same, and the degree of reinforcement compared to the control samples ranges from 32.9% for EK1 to 38.7% for EK3. The module of elasticity in all samples was increased by about 50% compared to the control sample, and the largest increase in the module of elasticity was achieved in sample EK3, which is 54.7%. Although the samples made of reinforced panels EK3 showed the greatest percentage improvement in ultimate load capacity, bending strength, and module of elasticity, the percentage increase is negligible compared to reinforced panels EK1 (about 5% for $F\;\mathrm{and}\;\sigma_{m∥d}$). However, the production of EK3 panels requires twice as much FRP material as EK1 panels. Given that the additional layers of carbon fabric are oriented perpendicular to the bearing direction and are not meant to accept the bending loads, these results are expected. From the aspect of increasing the edgewise bending strength and the module of elasticity, it is not economical to make reinforced panels EK2 and EK3. A comparative analysis of bending strength and module of elasticity according to reinforced combinations is shown in Figure 19.

Setting carbon fibers in the longitudinal direction reduced the variability of the bending results and contributed to a more consistent behavior of the structure. In addition, it is noticeable that the test samples from reinforced panels underwent much less deformation at much higher forces compared to the test samples from unreinforced ones, which is primarily contributed by carbon fibers and their behavior in the tensioned section zone, where the sudden brittle fracture occurs in unreinforced samples.

#### 3.2.2. Melamine Urea Formaldehyde Adhesive (MK) Samples

Although the test results of samples bonding with melamine-urea formaldehyde adhesive will not be considered due to the delamination of the test samples during testing, it must be noted that the control panels glued with this adhesive show a 6.96% higher value of bending strength and a 16.28% higher value of the module of elasticity compared to the control panels bonded with epoxy adhesive. The results of the test samples from the control panels, glued with MUF and MK4, are shown in Table 4.

### 3.3. Summary

According to the findings of the tests, the following can be summarized:

1. The percentage of improvement in bending strength for panels of type EK1 = 32.9%, EK2 = 33.2%, and EK3 = 38.7% compared to control panels, while the percentage of increase in modulus of elasticity is 54.1%, 50.7%, and 54.7% for EK1, EK2, and EK3, respectively. $E_{\|,\;EK1}=54.1\%,\;E_{\|,\;EK2}=50.7\%,E_{\|,\;EK3}=54.7\%;$
2. The fracture of all reinforced and unreinforced test samples glued with epoxy adhesive occurred as a brittle fracture in the tensile zone of the section;
3. When the maximum critical force was applied in the upper, compressed zone of the section, the test sample was crushed, the wood fibers were compressed, and to a small extent, the FRP-wood layers delaminated; the fracture occurred due to compressive stress;
4. In all test samples made of reinforced epoxy panels, the fracture in the wood was explosive, brittle, and mostly accompanied by the tearing of a smaller number of carbon fibers in the tension zone of the section;
5. In the case of test samples made from EK3 panels, delamination does not occur due to exceeding the compressive strength, and fiber breakage occurs only in a few test samples and to a much lesser extent;
6. The zone of plastic behavior before fracture in the test samples from EK3 panels is almost negligible. For specific test samples, the load intensity decreases after breaking, then increases again, and then permanently decreases;
7. Panels EK2 and EK3 reinforced with fibers set in two directions may have an advantage for other types of loading, especially for improving shear strength or pressure in the plane of the panel, perpendicular to the grain;
8. Carbon fibers oriented in the longitudinal direction, i.e., in the direction of the grain, affect the reduction of the variability of the results during bending and contribute to a more uniform behavior of the structure;
9. Carbon fibers oriented parallel to the grain, united through the pressed and tensioned zone of the section during bending, do not affect the shape of the fracture. In both reinforced and non-reinforced test samples, the fracture is brittle in the tensioned zone. However, when it comes to non-reinforced test samples, the fracture is explosive, sudden, and complete, splitting the sample in two;
10. Adhesion failure that occurred in the stressed zone between the FRP-wood and FRP-FRP layers in the test samples made of reinforced panels glued with epoxy adhesive occurred exclusively after the fracture in the wood;
11. During the testing of panels made of MUF adhesive, before the test sample breaks due to excessive force, delamination of the test sample occurs between the FRP-FRP layers in MK3 panels or the FRP-wood layer in MK1 panels;
12. Control panels glued with MUF with this adhesive show higher values of bending strength and modulus of elasticity compared to control panels glued with epoxy adhesive. This indicates that water-based glue is more favorable for gluing the wood itself due to its better penetration into the wood layer. However, due to the adhesion between FRP and wood as well as FRP-FRP glue, MUF is unsuitable for application.

## 4. Conclusions

In this study, laminated veneer lumber and reinforced laminated veneer lumber were produced with poplar veneer bonded with epoxy adhesive (EK) or melamine-urea formaldehyde adhesive (MK). Reinforced laminated veneer lumber was produced by inserting woven carbon fiber between veneers, and some of their mechanical properties were tested. Based on the presented results, the following conclusions were drawn:

- MUF adhesive is unsuitable for gluing synthetic fibers to each other or synthetic and wood fibers, and in veneer-veneer adhesion it is stable, but then breakage occurs on the wood during tests. Epoxy adhesive is applicable in all tested combinations.
- By reinforcing the LVL with carbon fibers and using epoxy adhesive, higher values of edgewise bending strength and modulus of elasticity are achieved.
- The edgewise bending strength and modulus of elasticity of reinforced LVL were significantly greater than those of unreinforced LVL, and the percentage of improvement for the EK3 combination are 38.7% and 54.7%, respectively. However, the most optimal strengthening from the aspect of material consumption and improvement of mechanical characteristics was achieved with reinforced panels of type EK1.
- All tested reinforced samples suffered much less deformation at much higher forces compared to unreinforced ones, which was contributed by the carbon fibers and their behavior in the tensioned section zone, where the sudden brittle fracture occurs in unreinforced samples.

The conducted research confirmed the possibility of applying the newly obtained poplar veneer composite material for the design of structural elements in LVL using epoxy adhesive. The application potential of the newly formed material tested through the work is reflected in the elements subjected to edgewise bending, i.e., linear beam elements or elements of polygonal shells and folded structures.

The study presents the results of some laboratory experiments conducted with samples of an indicative small scale. It is suggested that future studies address reinforcement with woven carbon on full-scale samples and test more mechanical properties.

## Figures and Tables

**Table 1:** Epoxy and MUF adhesive properties.

Technical Properties	Epoxy [34]		MUF [35]	
	Resin—A	Hardener—B	Resin—A	Hardener—B
Density ρ (20 °C) (g/cm^3^)	1.05	1.12	1.28	1.07
Brookfield viscosity (mPa·s (25 °C))	17,000	110	10,000–25,000	1700–2700
Dry matter content (%)			67.80	
Gel time (at 100 °C) (s)	110		45	

**Table 2:** Physical and mechanical properties of MapeWrap C UNI-AX 300.

Technical Properties	MapeWrap C UNI-AX 300 [37]
Mass (g/m):	300
Density (kg/m^3^):	1800
Equivalent thickness of dry fabric (mm):	0.164
Load-resistant area per unit of width (mm^2^/m):	164.3
Tensile strength (MPa)	≥4900

**Table 3:** Mechanical properties of RLVL and LVL in edgewise directions, epoxy adhesive.

Combination	u (mm)	F (N)	SD (%)	CoV (%)	$f_{m,0,edge}$ (N/mm^2^)	E (N/mm^2^)	SD (%)	CoV (%)
EK1	13.68	1828.56	99.52	5.44	97.71	12,214.47	601.04	4.92
EK2	12.18	1923.69	106.92	5.56	97.98	11,943.99	497.70	4.17
EK3	12.42	1924.63	73.45	3.82	101.97	12,256.66	461.63	3.77
EK4	15.93	1299.88	150.91	11.61	73.54	7925.08	280.80	3.54

u—mean of deflection, F—mean of bending force, SD—standard deviation, CoV—coefficient of variation, $f_{m,0,edge}$—edgewise bending strength, E—mean of the module of elasticity.

**Table 4:** Mechanical properties of LVL in edgewise directions, epoxy adhesive.

Combination	u (mm)	F (N)	SD (%)	CoV (%)	$f_{m,0,edge}$ (N/mm^2^)	E (N/mm^2^)	SD (%)	CoV (%)
MK4	12.87	1123.47	100.13	8.91	78.66	9215.47	1021.49	11.08

u—mean of deflection, F—mean of bending force, SD—standard deviation, CoV—coefficient of variation, $f_{m,0,edge}$—edgewise bending strength, E—mean of the module of elasticity.

## Data Availability

Not applicable.
